# Enfortumab vedotin therapy complicated by bullous dermatitis: A case report

**DOI:** 10.1016/j.jdcr.2025.03.025

**Published:** 2025-04-16

**Authors:** Roxana A. Hojjatie, Haya S. Raef, Taha O. Mohammed, Emily Henkel, Zachary Wolner

**Affiliations:** Department of Dermatology, Emory University School of Medicine, Atlanta, Georgia

**Keywords:** adverse drug reaction, autoimmune blistering diseases, bullous dermatitis, enfortumab

## Introduction

Antibody-drug conjugates are a class of targeted cancer therapies that combine a monoclonal antibody with a cytotoxic drug to selectively destroy tumor cells.^1^ Enfortumab vedotin (EV) is an antibody-drug conjugate used for the treatment of advanced urothelial carcinoma.^2^ EV targets cells expressing nectin-4d, disrupting microtubule networks, leading to apoptosis. Numerous adverse cutaneous reactions associated with EV have been reported, ranging from Stevens-Johnson syndrome/toxic epidermal necrolysis (SJS/TEN) to an exanthematous drug eruption.^3^ While these reactions usually resolve within days to weeks after discontinuation of the therapy,^4^ we present a unique case of bullous dermatitis in a patient undergoing EV treatment that persisted for several months after treatment discontinuation.

## Case description

A 78-year-old woman with a past medical history of type 2 diabetes mellitus, stage IV chronic kidney disease, and invasive high grade urothelial carcinoma on EV presented to the dermatology clinic with a several-day history of pruritic bullae on the lower extremities. She was diagnosed with urothelial carcinoma 2 years prior and her cancer was refractory to chemotherapy and pembrolizumab. She was transitioned to EV 15 (1.25 mg/kg) months prior to her dermatology visit, with excellent response. In clinic, she reported that the bullae initially developed on her feet and rapidly spread up her legs. She tried triamcinolone, hydroxyzine, diphenhydramine, and cetirizine with no improvement in the pruritus or blisters.

**Fig 1:**
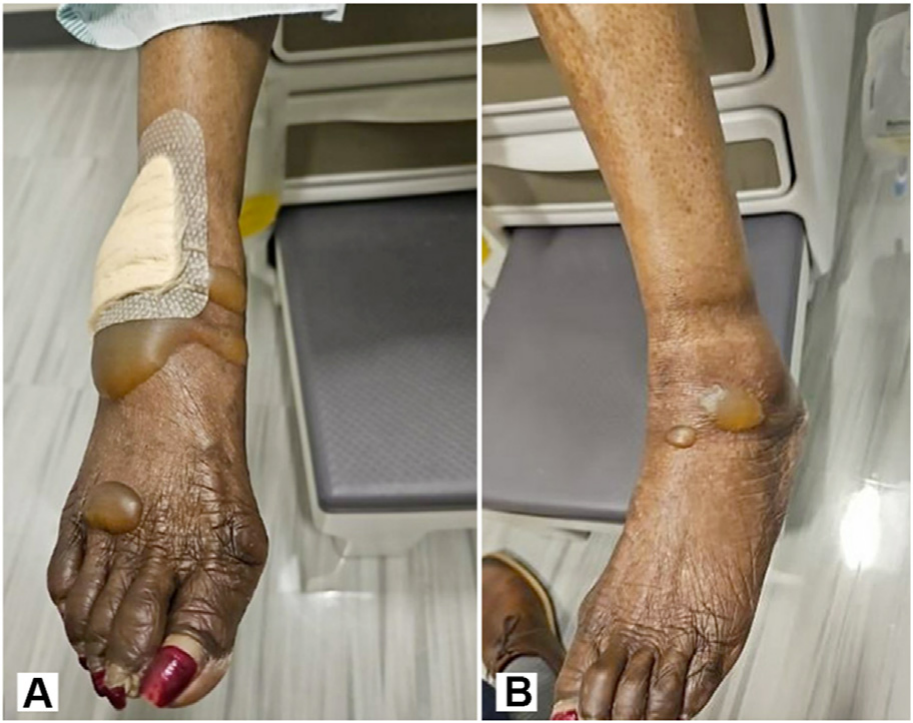
**A,** Initial patient presentation. On the right anterior ankle and dorsum of the foot, there are tense bullae. **B,** On the left anterior ankle, there are tense bullae.

**Fig 2:**
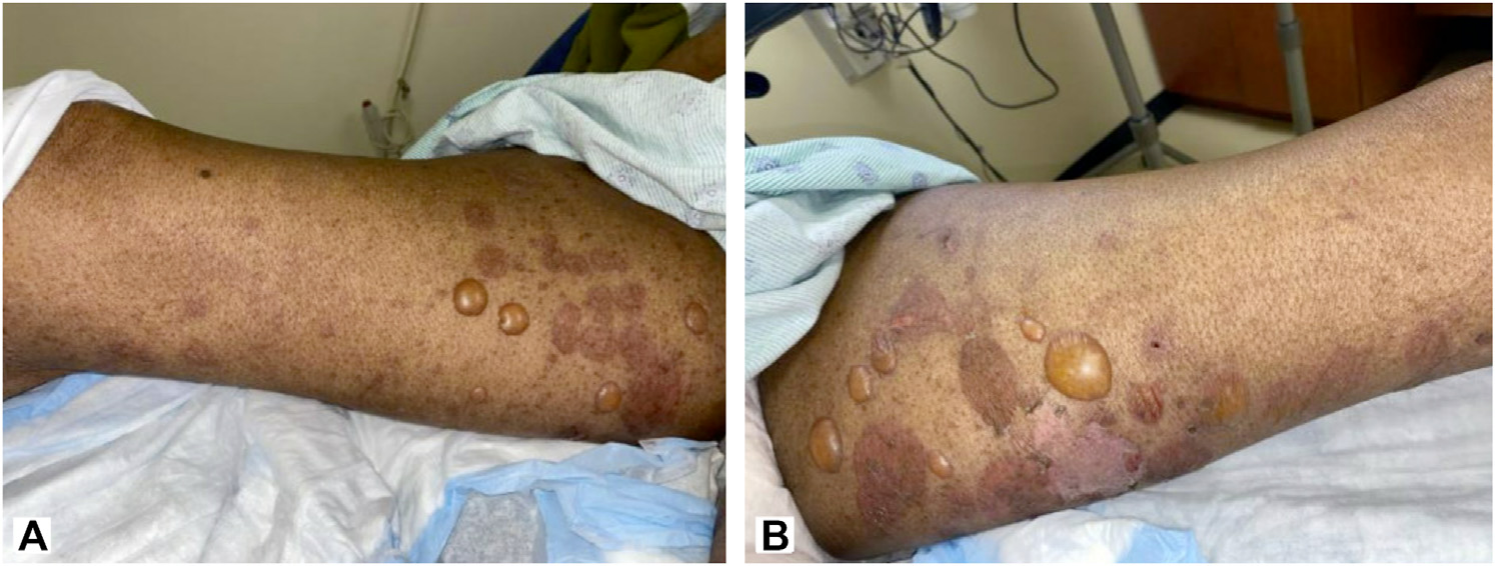
**A,** Patient presentation 11 days after the initial visit. On the right medial thigh, there are erythematous plaques, tense bullae, and scar-like plaques. **B,** On the left medial thigh, there are erythematous plaques, tense bullae, and scar-like plaques.

**Fig 3:**
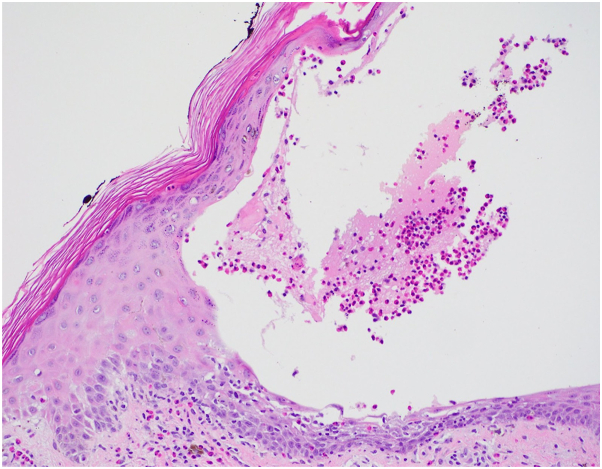
Intraepidermal bullae with subjacent dermal mixed inflammatory infiltrate containing eosinophilic spongiosis at the cleavage plane of the stratum spinosum (H&E, original magnification 20×).

Physical examination revealed numerous tense bullae of various sizes affecting the dorsal feet, legs, and thighs (Figs 1 and 2). Examination of the oral mucosa was unrevealing. Biopsy demonstrated intraepidermal bullae with subjacent dermal mixed inflammatory infiltrate containing eosinophilic spongiosis (Fig 3). Direct immunofluorescence of perilesional skin found scattered cytoid bodies with IgM. Indirect immunofluorescence on salt-split-skin was negative for IgG. Enzyme-linked immunosorbent assay was negative for BP180, BP230, and anti-desmogleins 1 and 3.

Based on the histopathologic and immunofluorescent findings, a diagnosis of bullous dermatitis secondary to EV was made. EV was held and her pruritus and bullae improved with a systemic steroid taper (40 mg taper over 20 days) and high-potency topical corticosteroids. Bullae and pruritus recurred once the prednisone taper was completed. She was subsequently transitioned to doxycycline 100 mg twice daily and niacinamide 500 mg twice daily for 1 week, dapsone 100 mg once daily for 8 weeks, and mycophenolate mofetil 500 mg twice daily 6 weeks, all without significant improvement.

Repeat enzyme-linked immunosorbent assay and indirect immunofluorescence were conducted at 8 and 16 weeks after initial testing and was negative for BP180, BP230, and desmogleins 1 and 3. Repeat direct immunofluorescence testing 20 weeks after initial testing only highlighted cytoid bodies. Given progression of her urothelial carcinoma on recent imaging, she was restarted on EV with a dose reduction (1 mg/kg). While her bullous dermatitis was initially stable, her bullous dermatitis disease returned with a grade 3 reaction requiring interruption of EV. Dupilumab was started with significant improvement in her pruritus and marked reduction in the number and size of her bullae. EV was ultimately restarted without flaring her bullous dermatitis.

## Discussion

Cutaneous adverse events following the initiation of EV are well-documented. In a combined analysis of 680 patients from EV clinical trials, 55% experienced skin reactions.^3^ While the exact mechanism behind skin toxicity associated with EV remains unclear, it is suspected to involve the drug's inhibitory effects on nectin-4 expression, a molecule crucial for epithelial cell-cell attachment that is present in both the skin and epithelial malignancies, such as urothelial carcinoma.^3^

The most frequent reported cutaneous reactions in EV trials included maculopapular rash (23%) and pruritus (33%).^5^ Serious cutaneous adverse effects (Grade ≥3) occurred in 13% of patients and included various reactions such as symmetrical drug-related intertriginous and flexural exanthema, bullous dermatitis, exfoliative dermatitis, and palmar-plantar erythrodysesthesia syndrome.^5^ Severe cutaneous adverse reactions have also been reported outside of the clinical trial setting, including erythema multiforme-like rash, lichenoid eruption and SJS/TEN.^6^, ^7^, ^8^

In our case, the patient developed bullous dermatitis, a reaction that has been noted in both clinical trials and postmarketing reports for EV. A recent case series described severe blistering eruptions resembling SJS/TEN secondary to EV, though without hallmark features such as full-thickness epidermal necrosis on histopathology.^4^ Although our patient displayed an autoimmune-like blistering eruption with pruritic bullae and characteristic histological features, immunofluorescent studies and enzyme-linked immunosorbent assay did not identify a pathologic autoantibody. It is possible that in our case, EV is directly binding to nectin-4 in the skin and disrupting epithelial adhesion. Other cases have also demonstrated negative immunofluorescence, suggesting that EV itself may act as an autoantibody against the function of nectin-4 in the skin.^4^^,^^9^

Our case is also unique in that the patient’s bullous dermatitis presented more than a year after beginning EV infusions, continued to persist for several months despite discontinuation of EV, and was refractory to multiple therapeutic interventions including topical corticosteroids, doxycycline, dapsone, and mycophenolate mofetil. This contrasts from reported cases where cutaneous reactions typically resolved within a few weeks to months with the use of corticosteroids and/or cessation of therapy. Of note, a recent case report demonstrated success in using dupilumab to manage a bullous drug eruption that resulted from EV/pembrolizumab.^10^ Given these findings, we have initiated dupilumab in our patient with significant improvement in her existing flare and prevention of additional flares when she resumed her EV therapy. It is possible that in our case, nectin-4 disruption led to an ongoing cycle of inflammatory signaling, months after her last infusion, leading to persistent blister formation and pruritus. While other potential diagnoses, such as multifocal fixed drug eruption, were considered, a triggering medication could not be identified, and the histopathology was incongruent.

Our findings add to a growing body of evidence that EV can provoke diverse dermatologic reactions with an unpredictable nature, including bullous dermatoses. The mechanisms behind these reactions remain poorly understood. It is important for dermatologists to be aware of the potential for skin manifestations as side effects of EV, as the drug is also being investigated for use in other cancers such as pancreatic carcinoma and penile squamous cell carcinoma. Additionally, the prolonged and refractory disease course in some patients, even after cessation of the drug and multiple interventions, underscores a need for further investigation into EV’s long-term effects on skin integrity.

## Conflicts of interest

None disclosed.
